# Dual effects of indoxyl sulfate on modulation of human hepatic CYP3A activity, with individual differences

**DOI:** 10.1371/journal.pone.0328182

**Published:** 2025-07-10

**Authors:** Masao Togao, Naoyuki Asakawa, Gaku Wagai, Yuki Ohta-Takada, Jun Otsuka, Minoru Ando, Akinobu Kurita, Koji Kawakami

**Affiliations:** Safety Research Department, Yakult Central Institute, Kunitachi-shi, Tokyo, Japan; Babasaheb Bhimrao Ambedkar University (A Central University), INDIA

## Abstract

This study aimed to identify gut microbiota-derived metabolites governing the activity of hepatic CYP3A in blood level. Indole propionic acid (IPA) and lithocholic acid, ligands of the pregnane X receptor, a transcriptional regulator of CYP3A, and various gut microbiota-derived metabolites in blood level were analyzed. Results revealed that IPA and lithocholic acid did not affect CYP3A activity, while indoxyl sulfate (IS), a uremic toxin, affected CYP3A across different cell lines. The effects of IS on primary hepatocytes from three donors were analyzed, and a concentration-dependent impact was observed, as the CYP3A activity decreased in one donor and increased in another. These findings offer initial insights into blood-level gut microbiota-derived metabolites influencing hepatic CYP3A. Furthermore, the study demonstrates that the response to IS, beyond its concentration, can cause variations in hepatic CYP3A activity among individuals. This study advocates accounting for the dual effects of IS and the benefits of personalized medicine.

## Introduction

Recent advancements in gut microbiota analysis have uncovered the multifaceted interactions between pharmaceutical agents and the gut microbiota, highlighting their intricate relationship [1–4]. The role played by the gut microbiota in modulating the host’s drug-metabolizing enzymes and the consequent impact on the metabolism of various drugs has garnered substantial attention [5–8]. Specifically, hepatic cytochrome P450 (CYP) 3A, a pivotal drug-metabolizing enzyme, has been observed to exhibit diminished activity in the absence or depletion of gut microbiota, both *in vitro* and *in vivo* [9–13]. Furthermore, our previous study has demonstrated that individual variations in gut microbiota substantially influence hepatic CYP3A activity and gene expression in human flora-associated mice models [14]. These findings highlight the fact that the variations in gut microbiota composition among individuals can alter the metabolism, efficacy, and toxicity of drugs metabolized by CYP3A.

Gut microbiota is believed to influence hepatic CYP3A modulation by delivering gut microbiota-derived metabolites to the liver, and subsequently regulate transcription by interacting with transcriptional regulators in hepatocytes [15]. Indole propionic acid (IPA) and lithocholic acid have been noted to be potential gut microbiota-derived metabolites capable of affecting CYP3A [9,16] through their interaction with a critical transcriptional regulator, the pregnane X receptor (PXR) [17]. Moreover, drug-metabolizing enzymes, including Cyp, contribute to the metabolism of tryptophan-derived indole to the uremic toxin indoxyl sulfate (IS) [18]. Conversely, IS has been reported to mildly impede CYP activity, including that of CYP3A, by directly interacting with CYP proteins at extremely high doses [18]. However, the specific gut microbiota-derived metabolites responsible for regulating hepatic CYP3A activity in the blood level have not yet been identified. Identifying these metabolites holds the potential of deciphering personalized medication and pioneering treatments mediated by the gut microbiota.

The present study aims to identify gut microbiota-derived metabolites that modulate hepatic CYP3A activity. The effects of metabolites on CYP3A activity were evaluated using two human hepatocyte cell lines and primary human hepatocytes. Our findings revealed that CYP3A activity in either cell line was not affected by both IPA as well as lithocholic acid. However, IS was observed to exhibit a previously unrecognized dual influence on CYP3A activity, exerting positive as well as negative effects across different cell lines and individual variations. Notably, this study offers initial insights into blood-level gut microbiota-derived metabolites influencing hepatic CYP3A. Furthermore, this study also demonstrated that, apart from IS levels, individual reactivity to IS might be responsible for the observed variations in CYP3A capacity.

## Materials and methods

### Chemicals

For the cellular assay, indole metabolites, such as IPA and IS, secondary bile acids (lithocholic and deoxycholic acids), and butyric acid, which has been suggested to affect several CYPs [13], were selected. Indole and dimethyl sulfoxide (DMSO) were procured from Fujifilm Wako Pure Chemicals (Osaka, Japan). DMSO served as the vehicle control. Rifampicin, omeprazole, and phenobarbital, served as positive controls for inducing CYP3A, CYP1A, and CYP2B, respectively, and were procured from Fujifilm Wako Pure Chemicals. IPA, indoleacetamide, and IS were procured from Sigma-Aldrich (St. Louis, MO, USA). Kanto Chemical (Tokyo, Japan) supplied the indoleacetic acid, secondary bile acids (lithocholic acid and deoxycholic acid), and butyric acid. Ultrapure water was obtained from Promega (Madison, WI, USA). Test compounds were prepared in accordance with previously reported human blood levels [19–23]. Concentrations for compounds lacking reported human blood levels, were standardized at 0.1 and 1 μM. Since IPA enhances the ligand capacity of PXR in the presence of indole [24], combination exposure including IPA and indole was also included.

The reagents including isopropanol and ethanol, which were necessary for RNA isolation, as well as the potassium dihydrogen phosphate solution utilized for microsome inhibition assays were obtained from Fujifilm Wako Pure Chemicals. Inhibitors of CYP3A4, CYP1A2, and CYP2B6, viz, ketoconazole, α-naphthoflavone, and clopidogrel sulfate, respectively, were obtained from Tokyo Chemical Industry (Tokyo, Japan) and were utilized as control compounds in a microsome inhibition assay [25]. The CYP2C9 inhibitor sulfaphenazole was obtained from Cayman Chemical (Ann Arbor, MI, USA) for the same purpose [25]. Ultrapure water served as the solvent for IS and phenobarbital, whereas DMSO was the solvent for the remaining substances.

### Cellular assay

All incubations were conducted at 37°C in a 5% CO_2_ incubator for the cell culture process, adhering to the manufacturer’s instructions.

#### DPX-2 cells.

The DPX-2 cells, a human PXR-expressing cell line derived from HepG2, a human hepatoma-derived cell line, were procured from Puracyp Inc. (Carlsbad, CA, USA). Upon receipt, the DPX-2 cells were thawed and suspended in culture media (Puracyp). A portion of the cell suspension was then mixed with an equal volume of 0.4% trypan blue solution for cell counting purposes. Approximately 5 × 10^4^ cells were dispensed into individual wells of a 96-well collagen I-coated plate and incubated overnight. After aspirating the incubated medium, medium containing the test substance adjusted to the appropriate concentration was added. The cells were then incubated for 48 h.

CYP3A activity was measured using the P450-Glo™ CYP3A4 Assay with Luciferin-IPA (Promega). After removal of the medium, 50 μL of assay medium containing the luminescent substrate was added to each well and was incubated for 1 h. Subsequently, the assay medium in each well was transferred into a white 96-well plate. After dispensing Luciferin detection reagent (50 μL) into each well of the white 96-well plate, the mixture was incubated at room temperature (21°C–25°C) for 20 min. A luminometer was then used to measure the Luminescence. The values were calculated by subtracting that of the blank control.

CellTiter-Fluor (Promega) was used to assess the cell viability. After dispensing the medium, 100 μL of CellTiter-Fluor reagent was added to each well of a 96-well plate, and the cells were incubated for 1 h. Fluorescence measurements were then obtained using a plate reader with excitation and fluorescence wavelengths set at 390 and 505 nm, respectively. The values were calculated by subtracting that of the blank control.

#### HepaRG^®^ cells.

HepaRG^®^ cells, human hepatocellular carcinoma cells, were procured from KAC Co., Ltd. (Kyoto, Japan). The preculture and assay media for HepaRG^®^ cells consisted of thawing/plating/general-purpose medium and serum-free induction medium (KAC). After procurement, HepaRG^®^ cells were thawed and suspended in a thawing/plating/general-purpose medium. After mixing and centrifugation, cell counting was performed using a trypan blue solution. Approximately 7.2 × 10^4^ cells were added to each well of a 96-well collagen I-coated plate and incubated for 72 h. After the incubation period, the medium was aspirated, and then medium containing the test substance adjusted to the appropriate concentration was added. The cells were incubated for 48 h and the medium was replaced once at 24 h with a new test substance-containing medium.

CYP3A activity was assessed using the same method employed for DPX-2 cells. CellTiter-Glo (Promega) was used for determining the cell viability. After measurement of CYP3A activity, 25 μL of CellTiter-Glo reagent was thoroughly mixed into each 96-well plate containing the cultured cells. 40 μL of the contents from each well were transferred into a new white 96-well plate. After incubation of the plate at room temperature for 10 min, luminescence was measured using a luminometer. The values were calculated by subtracting that of the blank control.

### Primary human hepatocytes

Inducing cryopreserved human hepatocytes (Plateable) (Xenotech, Lenexa, KS, USA) from three donors were utilized independently in this study. The cells were thawed and seeded using OptiThaw and OptiPlate media (Xenotech). The cells were counted and 5 × 10^4^ cells were dispensed into 96-well collagen I-coated plates. The cells were then incubated for 2–4 h, the medium was removed, and OptiCulture medium was introduced, followed by incubation for 24 h. After incubation, an assay medium containing the test substance was added and mixture was again incubated for 48 h. Medium replacement was conducted once at 24 h with a fresh test substance-containing medium for CYP3A activity measurement and RNA extraction.

CYP3A activity and cell viability were measured using the same method employed for HepaRG^®^ cells. RNA was extracted using the ReliaPrep™ RNA cell miniprep systems (Promega). The RNA purity was ensured by confirming that the A260/A280 ratio within the range of 1.8–2.2, followed by cDNA synthesis from the extracted RNA. Real-time polymerase chain reaction amplification of cDNA was conducted using the PowerUp SYBR Green Master Mix (Applied Biosystems, Waltham, MA, USA). Target genes included *CYP3A4*, *CYP1A2*, *CYP2B6*, *CYP2C9*, *PXR*, and its downstream gene *MDR1* [17]. The sequences of each primer have been provided in S1 Table. Analysis was performed using the ΔΔCt method, with glyceraldehyde-3-phosphate dehydrogenase (GAPDH) as the internal standard.

### CYP Inhibition assay

The P450-Glo™ CYP assay (Promega) was used for evaluating the direct inhibitory effect of IS on CYP3A4, CYP1A2, CYP2B6, and CYP2C9 activity with 50 pooled human liver microsomes (Xenotech), in accordance with the manufacturer’s instructions. After combining the microsomes, IS, and luminescent substrate at appropriate concentrations in 0.2 M potassium phosphate buffer (pH 7.4), the mixture was pre-incubated at 37°C for 10 min. The mixture was further incubated at 37°C for 10, 10, 20, and 30 min for CYP3A4, CYP1A2, CYP2B6, and CYP2C9, respectively after addition of NADPH regeneration systems solution (Promega). The solution was stabilized at room temperature for 20 min after addition of luciferin detection reagent. Luminescence was measured using a luminometer. Inhibitors of CYP3A4, CYP1A2, CYP2B6, and CYP2C9, namely, ketoconazole, α-naphthoflavone, clopidogrel sulfate, and sulfaphenazole, were used as positive controls to confirm the proper functioning of the system. Ultrapure water and DMSO were used as vehicle controls for IS and positive controls, respectively.

### Ethics statement

Xenotech, a supplier of primary human hepatocytes and microsomes, acquires human tissues and organs through partnerships with non-profit organ procurement organizations. These partnerships are managed by human tissue and organ recovery and placement networks, which coordinate the distribution of human tissues and organs from organ procurement organizations to Xenotech. Approved protocols by the Human Subjects Committee of the University of Kansas School of Medicine detail the use of these tissues. Xenotech is exempt from further review due to the concealed identity of tissue donors and the public availability of the provided tissues.

### Statistical analysis and reproducibility

In the cellular assay (DPX-2cells, Hepa RG^®^ cells, and three primary human hepatocytes), cell viability was quantified as a percentage relative to the negative control, which was a solvent. A viability cut-off value was established at 70% of the negative control’s viability. Compounds were classified as cytotoxic if they exhibited cell viability below this threshold. In this study, none of the compounds exhibited a reduction in cell viability below the defined cut-off value. Cell viability served as a correction factor for CYP3A activity.

The values for CYP3A activity and gene expression (ΔCt) in the cellular assay, as well as CYP3A, CYP1A, CYP2B, and CYP2C activities in the CYP inhibition assay, were normalized as percentages relative to the negative control. The Williams test, a test used when dose dependence is assumed in pharmacological studies [26], was used to analyze data from three replicate wells in three separate plates. A concentration-dependent monotonic increase or decrease was assumed, with a significance level of 2.5% and a significant trend of 5.0%. The Bell Curve for Excel (Social Survey Research Information Co., Ltd., Tokyo, Japan) was used for performing the statistical analyses.

## Results

### CYP3A activity in screening assays using DPX-2 cells

**Fig 1:**
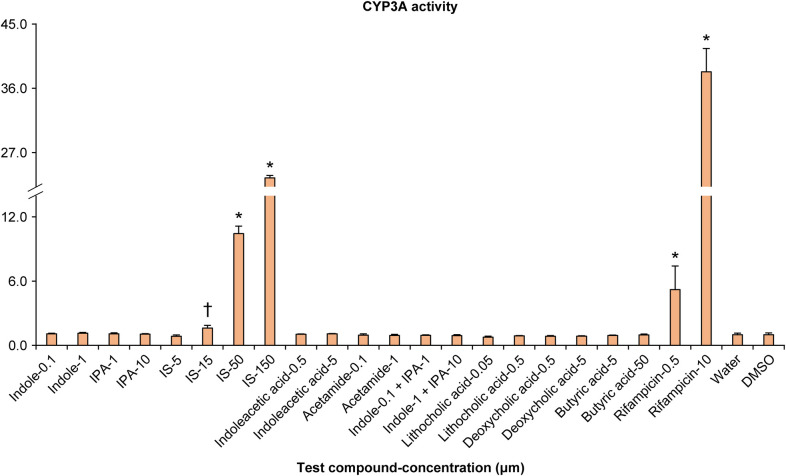
CYP3A activity in DPX-2 cells treated with each test compound. Data have been presented as CYP3A activity divided by cell viability and expressed as the ratio of the respective solvent (negative control). Water was used as the solvent for indoxyl sulfate (IS) and dimethyl sulfoxide (DMSO, final concentration 0.1%) was used as the solvent for the other compounds. The data represent the means ± SD from three plates. *: (P < 0.025); †: (P < 0.05).

The CYP3A activity in DPX-2 cells treated with each test compound has been depicted in Fig 1. IS notably enhanced CYP3A activity at 50 and 150 μM, with a noteworthy increase observed even at 15 μM. However, there were no substantial differences in CYP3A activity for other compounds, including IPA and lithocholic acid.

### CYP3A activity in screening assays using HepaRG^®^ cells

**Fig 2:**
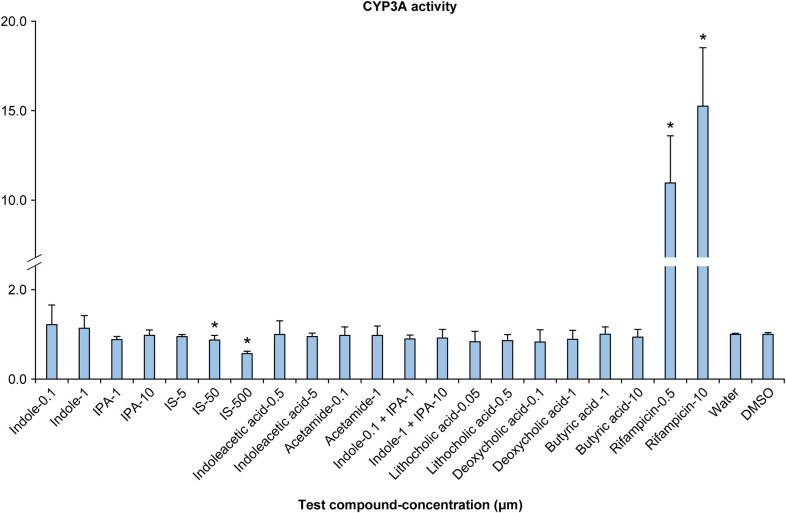
CYP3A activity in HepaRG^®^ cells treated with each test compound. Data have been presented as CYP3A activity divided by cell viability and expressed as the ratio of the respective solvent (negative control). Water was used as the solvent for indoxyl sulfate (IS) and dimethyl sulfoxide (DMSO, final concentration 0.1%) was used as the solvent for the other compounds. The data represent the means ± SD from three plates. *: (P < 0.025).

The CYP3A activity in HepaRG^®^ cells treated with each test compound has been illustrated in Fig 2. IS notably decreased CYP3A activity at 50 and 500 μM concentrations. However, the other compounds failed to elicit substantial differences in CYP3A activity.

### CYP3A activity and gene expression levels in human hepatocytes

**Fig 3:**
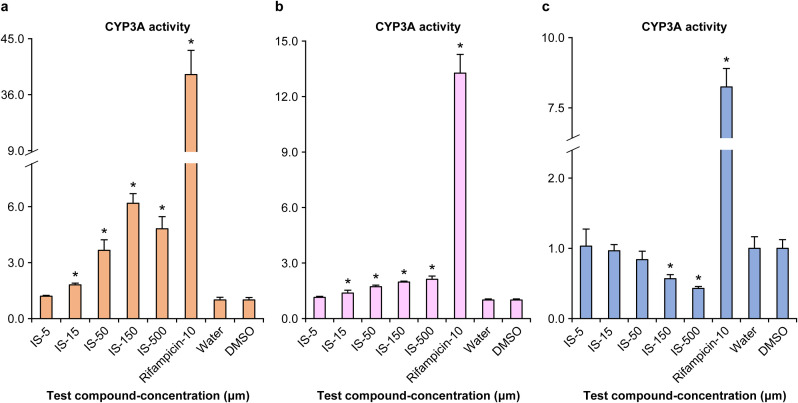
CYP3A activity in primary human hepatocytes treated with indoxyl sulfate (IS). Data have been presented as CYP3A activity divided by cell viability and expressed as the ratio of the respective solvent (negative control). Water was used as the solvent for IS and dimethyl sulfoxide (DMSO, final concentration 0.1%) was used as the solvent for rifampicin. The data represent the means ± SD from three plates. *: (P < 0.025). a, donor A; b, donor B; c, donor C.

Due to the conflicting results observed with IS exerting both positive as well as negative effects on CYP3A activity in DPX-2 and HepaRG^®^ cells, we opted to conduct a thorough investigation of these effects using primary human hepatocytes from three donors. As evident from Fig 3, the CYP3A activity was assessed in human hepatocytes treated with IS. IS induced a concentration-dependent increase in CYP3A activity in donors A and B, with statistically significant elevations noted at 15, 50, 150, and 500 μM. However, in donor C, CYP3A activity declined with increasing concentrations. Notably, statistically significant reductions were observed at 150 and 500 μM.

**Fig 4:**
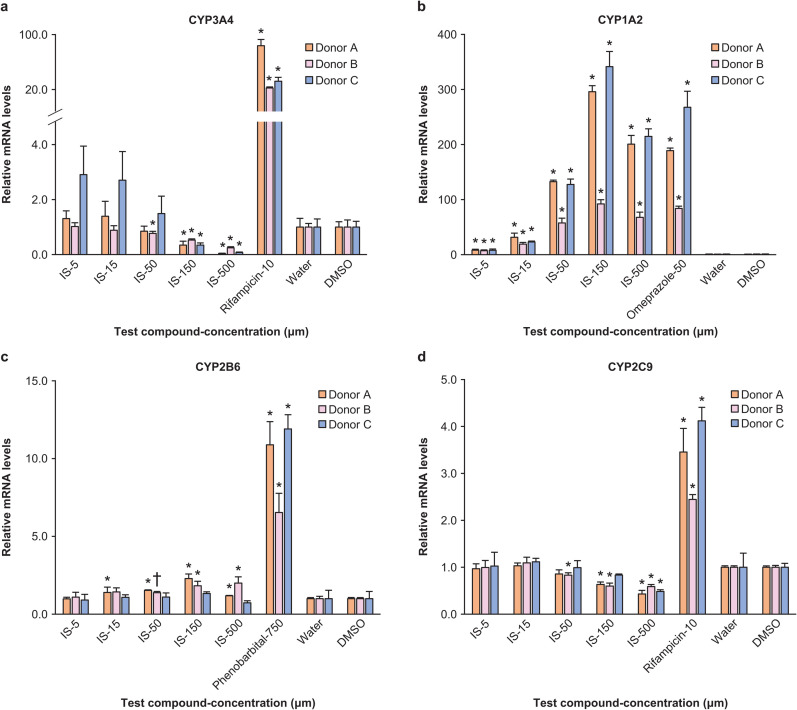
CYP gene expression of indoxyl sulfate (IS) in primary human hepatocytes. GAPDH (glyceraldehyde-3-phosphate dehydrogenase) was used as an internal standard, and the expression of each gene was expressed as the ratio of the respective solvent (negative control). Water was used as the solvent for the IS and phenobarbital, and dimethyl sulfoxide (DMSO, final concentration 0.1%) were used as the solvents for rifampicin and omeprazole. The data represent the means ± SD from three plates. *: (P < 0.025), †: (P < 0.05). a, CYP3A4; b, CYP1A2; c, CYP2B6; d, CYP2C9.

The expression levels of *CYP* genes were subsequently evaluated (Fig 4). IS decreased *CYP3A4* expression levels in a concentration-dependent manner in donors A and B, with substantial reductions observed at 150 and 500 µM in donor A and at 50, 150, and 500 µM in donor B. However, for donor C, a substantial reduction was observed at 150 and 500 µM, while an unexpected increase was observed at 5, 15, and 50 µM. In all donors, *CYP1A2* exhibited a concentration-dependent and substantial increase in gene expression in response to IS. *CYP2B6* demonstrated a concentration-dependent increase in donors A and B, with substantial increases observed at 15, 50, 150, and 500 µM for donor A and 150 and 500 µM for donor B. Meanwhile, substantial decreases were observed for *CYP2C9* at 150 and 500 µM in donor A, 50, 150, 500 µM in donor B, and 500 µM in donor C.

A distinct upregulation in *PXR* expression was observed at concentrations of 15, 50, 150, and 500 μM in donor C. Meanwhile, a substantial decrease was observed in the expression of multidrug resistance protein 1 (MDR1), a downstream gene regulated by *PXR*, at 50, 150, and 500 μM across all donors (S1 Fig).

### Direct CYP inhibition in human microsomes

Furthermore, using pooled human liver microsomes, we examined the direct inhibitory effect of IS on CYP, in accordance with the established guidelines [25]. The findings revealed that IS exhibited weak inhibition of CYP3A, CYP1A, CYP2B, and CYP2C activity (S2 Fig). Specifically, CYP3A displayed a downward trend, and CYP1A exhibited a substantial decrease at 500 μM, while CYP2B and CYP2C showed considerable decreases at all concentrations.

## Discussion

In this study, IS was identified to be a gut microbiota-derived metabolite capable of influencing CYP3A activity at human blood levels. We also deciphered the dual positive and negative impacts exerted by IS on CYP3A, which varied among individuals.

Initially, we concentrated on the gut microbiota-derived metabolites IPA [16] and lithocholic acid [9] that are known to bind to PXR and potentially stimulate CYP3A activity. However, no substantial increase in CYP3A activity was observed in any human hepatocyte cell lines exposed to IPA or lithocholic acid at concentrations corresponding to those in human blood levels. A couple of previously conducted studies had indicated that the concentrations required for IPA and lithocholic acid to enhance PXR activity were approximately 120 μM [16] and 10 μM [27], respectively, which were quite high compared with the concentrations used in this study, thereby indicating that the practical impact of these substances on hepatic CYP3A activity might be limited. This prompted us to focus on other metabolites for further evaluation.

DPX-2 cells, known for their utility in screening CYP3A inducibility [28], demonstrated a concentration-dependent increase in CYP3A activity following exposure to IS, thereby indicating that IS enhances CYP3A activity. To ascertain whether this effect is specific to the particular cell line, the CYP3A activity was re-evaluated using HepaRG^®^ cells, which enable assessment of the inducibility of various CYP isoforms, including CYP3A [29]. Interestingly, the results revealed that the CYP3A activity decreased in a concentration-dependent manner in HepaRG^®^ cells upon exposure to IS, which were contradictory to the effects observed in DPX-2 cells. This indicates that the effect of IS on hepatic CYP3A activity could be complex- and situation-dependent. Moreover, these findings highlight the limitations of relying on a single cell line for assessment. In drug development, primary human hepatocytes are the most accurate model for inferring human responses to CYP induction [25]. Guidelines for evaluating CYP inducibility in drug development recommend using cells from three or more donors to account for potential individual differences in responses [25]. Therefore, primary human hepatocytes from three donors were used in this study to verify the effect of IS on CYP3A activity. The results demonstrated a concentration-dependent increase in CYP3A activity in hepatocytes from two donors (donors A and B); however, a concentration-dependent decrease was observed in CYP3A activity in the hepatocytes from the third donor (donor C). While few studies reporting divergent positive or negative effects among donors are available, obeticholic acid, a synthetically modified bile acid has been reported to enhance CYP2B activity in primary human hepatocytes from one donor in a concentration-dependent manner while lowering CYP2B activity in hepatocytes obtained from another donor [30]. Therefore, the dual effect of IS on hepatic CYP3A observed in this study is plausible. The principles for conducting CYP induction studies in drug development indicate that if CYP activity is impacted in any of the three donors, the drug can potentially affect CYPs [25]. These findings indicate that IS affects CYP3A activity in multiple ways, and the manner and extent of this impact can vary among individuals. Furthermore, the results highlighted a substantial disparity in CYP induction at the maximum concentration between donors with elevated and diminished CYP3A activity, which varied approximately 10-times. In humans, individual differences in CYP3A protein expression and activity have been reported to vary to a great extent [31,32], which is associated with the unanticipated attenuation of drug efficacy and unexpected toxicity [33]. These results imply that the variability in CYP3A activity, drug efficacy attenuation, and toxicity expression might be substantially affected by the individual differences in the concentration of IS and reactivity to it.

Regarding the actual clinical relevance of the exposure concentrations in this study, in healthy individuals, the blood concentration of IS has been reported to typically be less than 10 μM [19]. However, the IS concentration in patients with end-stage chronic renal failure exceeds 15 μM, the minimum effective level in this study, with an average of approximately 250 μM and a maximum of approximately 550 μM [19]. Therefore, it can be considered that human exposure to IS substantially impacts hepatic CYP3A function in clinical practice. A previous study had demonstrated a positive correlation between serum IS concentrations and the need for higher doses of cyclosporine, a drug metabolized by CYP3A, to attain target blood concentrations in transplant recipients and individuals with chronic kidney disease [34]. Conversely, a negative correlation between blood IS levels and CYP3A activity in patients with renal diseases has been demonstrated by another study [35]. In a recent animal study, IS concentrations were positively correlated with the concentrations of the CYP3A-metabolized drug in male mice; i.e., the ability to metabolize the CYP3A-metabolized drug was negatively correlated with IS [36]. Additionally, other studies have reported a lack of any clinically significant association between IS concentrations and CYP3A [37,38]. Therefore, consistent conclusions about the relationship between IS and CYP3A have yet to be drawn. The current findings indicate that individual variations in reactivity to IS might have been a hindrance in understanding the correlation between blood IS concentration and CYP3A activity in previous studies [37,38]. In other words, the observed disparities in levels of CYP3A-metabolized drugs among individuals in clinical practice might be attributed to IS and its impact on CYP3A reactivity.

With regards to the CYP gene expression levels, a concentration-dependent decrease in *CYP3A4* gene expression was observed in all donors, including donors A and B at high concentrations. In contrast, increased *CYP3A4* expression was observed in donor C at lower concentrations. These findings contradict the CYP3A activity results described earlier. Discrepancies in the activities and expressions of CYPs are also common. For instance, a previously conducted study in mice has demonstrated reduced hepatic *CYP3A* gene expression during pregnancy while activity had increased [39]. Moreover, a discrepancy between CYP3A activity and gene expression in mice colonized with normal mice gut microbiota has also been previously observed by us [40]. Thus, this study validates that the discordance between CYP3A activity and gene expression observed in mice extends to human hepatocytes as well. With regards to the mechanism underlying the decrease in *CYP3A4* expression noted in this study, it is conceivable that the PXR system may be implicated in the reduction of *CYP3A4* gene expression, since the expression level of *MDR1*, a downstream gene of *PXR* akin to *CYP3A* [17], decreased in a concentration-dependent manner across all donors.

For CYP isoforms other than *CYP3A4*, IS prompted a concentration-dependent increase in gene expression for *CYP1A2*. A previous study had illustrated the role of IS as a ligand for the nuclear receptor, aryl hydrocarbon receptor, the primary regulator of *CYP1A2* [41]. The present study has confirmed the findings of the previous research. Furthermore, IS induced a concentration-dependent increase in *CYP2B6* expression and a concentration-dependent decrease in *CYP2C9* expression. No previous reports have documented the effect exerted by IS on *CYP2B6* or *CYP2C9* expression. These results indicate the possible impact of IS on not only CYP3A but also various other CYP isoforms and their associated metabolizing drugs.

Direct inhibition studies that had been conducted using human liver microsomes in accordance with the guidelines, revealed that CYP activity was mildly inhibited by the direct interaction of IS with CYP protein in an isoform-independent manner. A previous study has reported that CYP3A activity was inhibited by high IS levels interacting with CYP3A protein [42]. These findings indicate that the contribution of direct inhibition is minimal among the effects of IS on CYP3A activity.

Based on the findings of the present study, one potential approach toward achieving stable usage of CYP3A metabolizing drugs involves use of interventions targeting the gut microbiota for mitigating individual differences in hepatic CYP3A activity. IS may lead to the exacerbation of individual variations in hepatic CYP3A activity due to its dual effect on CYP3A activity, as described earlier. Certain probiotics and drugs have been reported to reduce IS concentrations in the bloodstream [43–46]. Thus, individual differences in hepatic CYP3A activity can be decreased by lowering IS levels through probiotics or drugs. This could potentially minimize the risk of unexpected reduction in drug efficacy and unforeseen toxicity, promoting stable utilization of drugs.

The principal limitation hindering the generalization of these findings is the small sample size of primary hepatocyte donors (n = 3) utilized, precluding speculation on the prevalence of positive and negative effects of IS within the human population. It seems reasonable that the primary hepatocytes employed in this study were subject to exceptionally unique conditions. Nevertheless, according to established guidelines, the evaluation of drug interactions in pharmaceutical development should use primary hepatocytes from three or more donors and the tested compounds can be considered CYP modulators if they influence primary human hepatocytes of even only a single donor [25]. Thus, we believe that the outcomes of this study are significant enough to recognize that IS has dual effects on CYP3A activity and to encourage future investigations involving large sample sizes to elucidate the prevalence of positive and negative effects of IS within the human population.

Several potential roadmaps should be considered to improve our understanding of the relationship between CYP3A regulation and gut microbiota. First, future studies should investigate the mechanism underlying the dual effects of IS observed in the present study to inform personalized medicine. For instance, gene polymorphisms are a major factor influencing individual differences in CYP activity [47,48] and include not only the polymorphisms in CYPs but also those in nuclear receptors, such as PXR [48,49]. It is possible that individual differences in IS response might be also due to such genetic polymorphisms. The contribution of these genes can be determined by evaluating the association between CYP activity and gene polymorphisms using a larger sample size. Second, although IS was the only metabolite found to modulate CYPs in the present study, uremic serum and other uremic toxins have been demonstrated to modulate CYP3A protein expression and activity [50,51]. In addition, uremic toxins have been shown to interact with IS. For instance, when added to serum samples, uremic toxins such as 3-Carboxy-4-methyl-5-propyl-2-furanpropionic acid, hippuric acid, and IS tended to induce CYP3A, albeit without statistical significance, in the serum-exposed LS180 cells, whereas exposure to a mixture of these metabolites reduced CYP3A expression in these cells [50]. Therefore, future studies should evaluate the effects of the interaction of IS with uremic toxins and other metabolites. Furthermore, it is likely that other metabolites besides IS impact CYP3A, including unknown metabolites as well as those that are relatively unknown. Thus, in-depth metabolomic profiling of comprehensive metabolites would be beneficial in identifying newer microbial metabolites that influence CYP3A.

In conclusion, IS, a gut microbiome-derived uremic toxin, has the potential to both increase and decrease CYP3A activity, with the direction and magnitude of these dual effects varying among individuals. It is expected that the findings of this study will prompt the consideration of optimizing drug usage by considering the gut microbiota, thereby advancing the field of personalized medicine and the development of innovative medical probiotics in the future.

## Supporting information

S1 Fig *PXR* and *MDR1* gene expression in indoxyl sulfate (IS)-exposed primary human hepatocytes. *GAPDH* serves as an internal standard, and gene expression is presented as the ratio compared with the respective solvent (negative control). Water was used as the solvent for IS, whereas dimethyl sulfoxide (DMSO) at a final concentration of 0.1% was used as the solvent for rifampicin. Data are presented as means ± standard deviation from three plates. *: (P < 0.025). a, *PXR*; b, *MDR1.*
(DOCX)

S2 Fig The inhibitory effect of IS on hepatic microsomal CYP activity. Data are presented as ratios of respective solvents (negative controls). Water was used as the solvent for IS, whereas DMSO at a final concentration of 0.1% was used as the solvent for the positive control. Data are presented as means ± standard deviation from three plates. *: (P < 0.025), †: (P < 0.05). a, CYP3A; b, CYP1A; c, CYP2B; d, CYP2C.
(DOCX)

S1 Table Sequences of real-time quantitative polymerase chain reaction primers used in the study. (DOCX)
