# Supplementary material for: Dual effects of indoxyl sulfate on modulation of human hepatic CYP3A activity, with individual differences
Source: PLoS One. 2025 Jul 10;20(7):e0328182. doi: 10.1371/journal.pone.0328182 (PMC12244530; doi:10.1371/journal.pone.0328182)
Supplement: S1 Fig — GAPDH serves as an internal standard, and gene expression is presented as the ratio compared with the respective solvent (negative control). Water was used as the solvent for IS, whereas dimethyl sulfoxide (DMSO) at a final concentration of 0.1% was used as the solvent for rifampicin. Data are presented as means ± standard deviation from three plates. *: (P < 0.025). a, PXR; b, MDR1. (DOCX) [file pone.0328182.s001.docx]

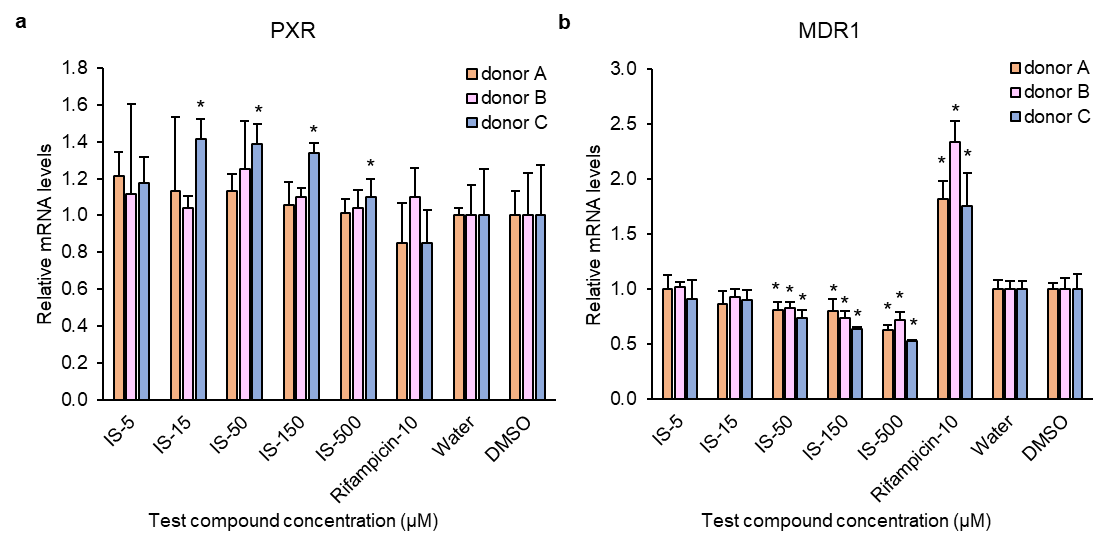


S1 Fig: PXR and MDR1 gene expressions of indoxyl sulfate (IS) in primary human hepatocytes. GAPDH served as an internal standard, and the expression of each gene was expressed in terms of the ratio with the respective solvent (negative control). Water was used as the solvent for IS, while DMSO (final concentration, 0.1%) was utilized as the solvent for rifampicin. The data represent the means ± SD from three plates. *: (P < 0.025). a, PXR; b, MDR1
