# Supplementary material for: Dual effects of indoxyl sulfate on modulation of human hepatic CYP3A activity, with individual differences
Source: PLoS One. 2025 Jul 10;20(7):e0328182. doi: 10.1371/journal.pone.0328182 (PMC12244530; doi:10.1371/journal.pone.0328182)
Supplement: S2 Fig — Data are presented as ratios of respective solvents (negative controls). Water was used as the solvent for IS, whereas DMSO at a final concentration of 0.1% was used as the solvent for the positive control. Data are presented as means ± standard deviation from three plates. *: (P < 0.025), †: (P < 0.05). a, CYP3A; b, CYP1A; c, CYP2B; d, CYP2C. (DOCX) [file pone.0328182.s002.docx]

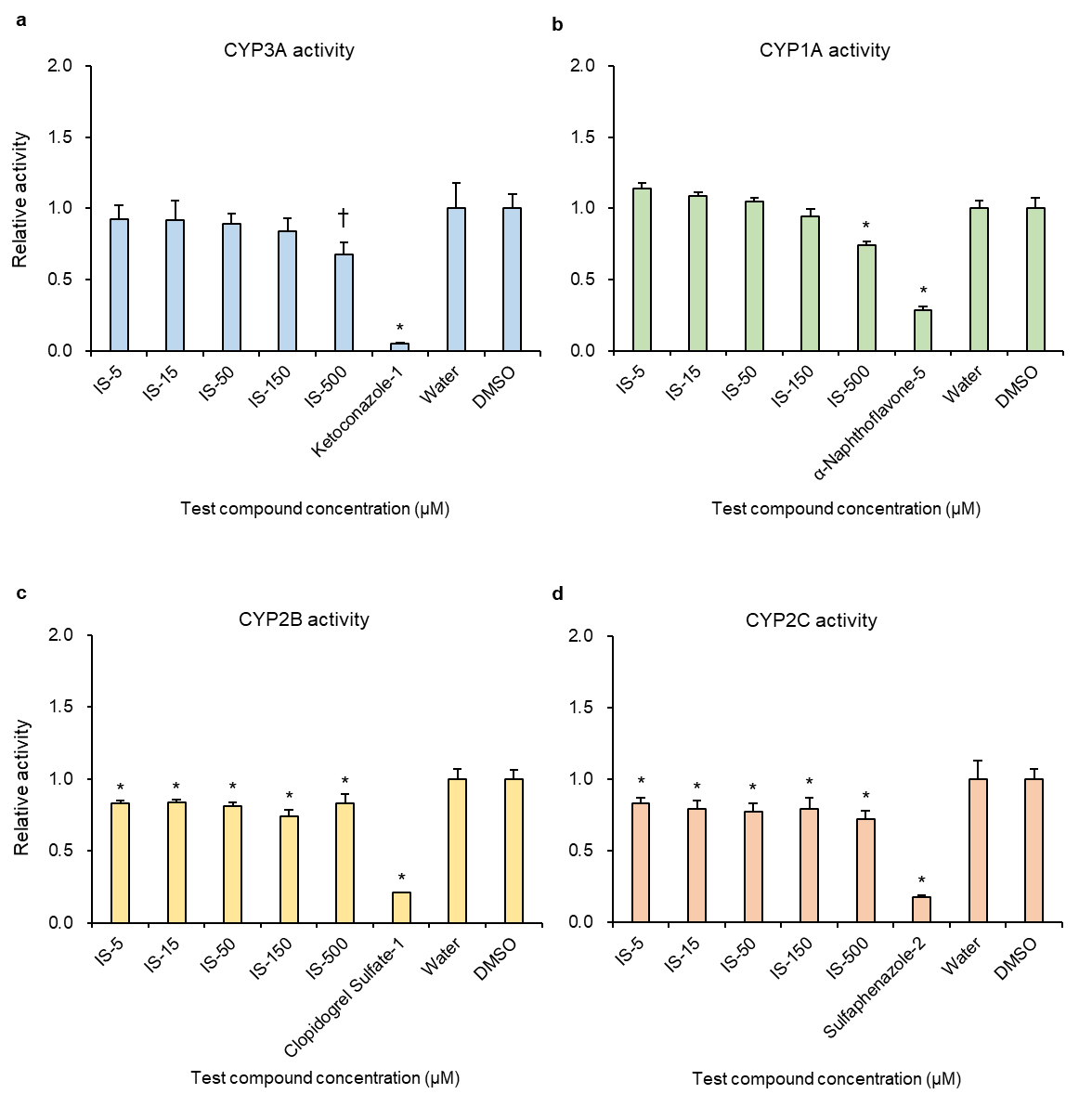


S2 Fig**.**: Inhibitory effect of IS on hepatic microsomal CYP activity. Data have been presented as ratios of respective solvents (negative controls). Water was used as the solvent for IS, while DMSO (final concentration, 0.1%) was utilized as the solvent for the positive control. The data represent the means ± SD from three plates. *: (P < 0.025), †: (P < 0.05). a, CYP3A; b, CYP1A; c, CYP2B; d, CYP2C
