# Supplementary material for: Dual effects of indoxyl sulfate on modulation of human hepatic CYP3A activity, with individual differences
Source: PLoS One. 2025 Jul 10;20(7):e0328182. doi: 10.1371/journal.pone.0328182 (PMC12244530; doi:10.1371/journal.pone.0328182)
Supplement: S1 Table — (DOCX) [file pone.0328182.s003.docx]

S1 Table: Sequence of RT-qPCR primers

| Primer | Forward (5’-3’) | Reverse (5’-3’) |
| --- | --- | --- |
| CYP3A4 | GCCTGGTGCTCCTCTATCTA | GGCTGTTGACCATCATAAAAG |
| CYP1A2 | CATCCCCCACAGCACAACAA | TCCCACTTGGCCAGGACTTC |
| CYP2B6 | TTCCTACTGCTTCCGTCTATCAAA | GTGCAGAATCCCACAGCTCA |
| CYP2C9 | AGCTTGGAAAACACTGCAGT | CCTGCTGAGAAAGGCATGAAG |
| PXR | TGCGAGATCACCCGGAAGAC | ATGGGAGAAGGTAGTGTCAAAGG |
| MDR1 | CCCATCATTGCAATAGCAGG | GTTCAAACTTCTGCTCCTGA |
| GAPDH | TGCACCACCAACTGCTTAGC | GGCATGGACTGTGGTCATGAG |
